# The effects of dairy products on seizure tendency in mice

**DOI:** 10.1016/j.heliyon.2019.e01331

**Published:** 2019-03-12

**Authors:** Soroor Inaloo, Fatema Pirsalami, Mona Dastgheib, Leila Moezi

**Affiliations:** a Neonatal Research Centre, Shiraz University of Medical Sciences, Shiraz, Iran; b Department of Pharmacology, School of Medicine, Shiraz University of Medical Sciences, Shiraz, Iran; c Pharmaceutical Sciences Research Center, Shiraz University of Medical Sciences, Shiraz, Iran

**Keywords:** Neuroscience

## Abstract

Epilepsy is a common neurological disorder which occurs as a result of a spontaneous electrical discharge in the brain. According to recent studies there might be a relationship between specific diet and seizure occurrence. Casein is an important protein of milk which often causes hypersensitivity. It seems the release of inflammatory cytokines during the process of immune system response alter the blood-brain-barrier (BBB) integrity and lead to neuronal inflammation which could constitute on epileptogenic focus. On the other hand, several studies represent full-fat milk or higher fat dairy products as an effective anti-inflammatory factor which elevate seizure threshold. The aim of present study was investigation of acute and chronic effects of dairy products including dough (a yogurt-based beverage), cheese, low and high fat yogurt and milk on pentylenetetrazole (PTZ)-induced seizures or electroshock in mice. The results of study indicated that lower fat dairy products reduced seizure threshold in intravenous PTZ-induced seizure as well as reduction in myoclonic and clonic jerk latencies in intraperitoneal PTZ-induced seizure. High fat products or cheese reduced seizure activity in both PTZ-induced models. Meanwhile both acute and chronic administration of dairy products had no effect on an electroshock-induced seizure. Therefore, diet-related seizures may depend upon the method which seizures are provoked.

## 1 Introduction

Epilepsy is one of the most common chronic disorders which affects 0.5–1% of general population [1]. It occurs as a result of uncontrolled and spontaneous electrical discharge in the brain [2]. Recent studies have reported there might be a relationship between consumption of specific foods or allergens and seizure occurrence which have increased general concerns among physicians and patients [3]. For example, excess dietary aminoacids induced seizure in rats and monosodium glutamate reduced the threshold of seizure [4, 5, 6]. Among different foods which may trigger the seizure occurrence, dairy products are major concerns because of excess use of a variety of them in dairy diet and several studies demonstrated cow's milk protein allergy which may induce epilepsy [7]. Although the exact molecular mechanism of epilepsy is still unknown, but it seems that central nervous system inflammation as a result of milk protein hypersensitivity may be responsible [8]. Indeed, the released inflammatory cytokines during the process of immune system response, cross the blood-brain-barrier (BBB) and may promote seizure susceptibility through neuronal inflammation [8]. Furthermore, the content of aminoacid-derived neurotransmitters may be altered by special diet such as dairy products. For instance opiate-like peptides which have been found in milk, are involved in the brain metabolism and they might play a crucial role in pathophysiology of epilepsy [9].

Recently, a large number of investigations emphasizes on the essential roles of dairy products on different aspects of human health. The advantages of milk products arise from their various components such as proteins, minerals, vitamins, lipids and carbohydrates or oligosaccharides which has strong effects on cognitive performances, control of blood pressure and diabetes or prevention from cardiovascular diseases [10, 11]. Although these beneficial effects are undeniable, in present study we evaluated the dairy products from another aspect.

The aim of present study was the evaluation of the acute and chronic effects of different dairy products including dough (a yogurt-based beverage), cheese, low and high fat yogurt and milk on the following: 1) myoclonic, clonic, andgeneralized tonic seizures and death induced by intraperitoneal administration of pentylenetetrazole (PTZ); 2) clonic seizure threshold induced by intravenous infusion of pentylenetetrazole (PTZ); and 3) tonic seizures induced by electroshock in mice. In the light of study results we may find is it necessary to restricts dairy products in a diet of epileptic patients or not.

## 2 Materials and methods

### 2.1 Animals

Male albino mice (25–35 g) were used in the study. They were housed in groups of 6 and were allowed free access to food and water. All animals were acclimated at least 3 days before all experimental were conducted during the period between 10.00 AM and 14.00 PM with normal room light (12 h regular light/dark cycle) and room temperature (23 ± 2 °C). All procedures were carried out in accordance with the institutional guidelines for animals’ care and use and all possible measures were taken to minimize the number of used animals. The procedure approved by Shiraz University of Medical SciencesEthics Committee prior to conducting our research (No: 95-01-36-13167). Each mouse was used only once and each treatment group consisted of 6–8 animals.

### 2.2 Dairy products

Dough (0.5% fat), low-fat yogurt (1.3 % fat), high-fat yogurt (4% fat), low-fat milk (1.5% fat), high-fat milk (3% fat) and cheese (% 40 fat in dried material)were used in this study which all purchased from Iran Dairy Industries Co. (Pegah).

### 2.3 Chemicals

Pentylenetetrazole (PTZ) is a GABA_A_ receptor antagonist [12] which was purchased from Sigma and it was prepared in saline as a 0.5% solution when administered through intravenous infusion.

### 2.4 Behavioral seizure evaluation

#### 2.4.1 Intraperitoneal PTZ-induced seizure

In this model, single intraperitoneal infusion of PTZ (85 mg/kg, CD 97) is used to induce a generalized tonic-clonic seizure. Immediately after PTZadministration, the animal was transferred to an open field (50 cm in diameter) and observed for 30 min for the occurrence of convulsion or death. Time latencies for the appearance of the first myoclonic jerk and generalized clonus were measured following PTZ administration. Latency is defined as the time between PTZ injection and the onset of myoclonic and clonic seizures. Myoclonus was described as sudden jerks. Generalized clonus was described as the involvement of all four limbs and tail rearing, wild running and jumping, sudden loss of upright posture, and autonomic sign such as hypersalivation and defecation. The incidence of tonic seizure and death was also recorded. Tonic seizure was defined as a full tonic hind-limb extension (THE) when the angle off the hind limbs to the torso exceeded 90° (usually 180°).

#### 2.4.2 Intravenous PTZ-induced seizure

In this model, a 4.6 mm diameter catheter was connected to the animal tail through 30-gauge dental needle and a syringe pump (Harvard, USA) was used to deliver an exact amount of PTZ with the rate of 0.5 mL/min through a catheter. The infusion was halted were forelimb clonus followed by full clonus of the body was observed. The minimal dose of PTZ (mg/kg of mice weight) needed to induce the clonic seizure, was considered as an index of seizure threshold.

#### 2.4.3 Electroshock test

To access the effects of dairy products in the modulation of susceptibility to an electroshock-induced seizure, an electroconvulsive therapy apparatus (Model 7800, Ugo Basile, Camerio, Italy) was used. By passing alternating current (50 Hz, 50 mA and 0.2 s) through ear electrodes, tonic convulsions of the mice hind extremities were induced. In order to improve electrode contact, the electrodes were moistened with normal saline before being attached to the ears of mice. Electroshock induces a continuum of motor convulsions which are dependent on the intensity of the electrical stimulation current. The current used was predetermined before experimentation and was the current that caused hind limb extension in all mice in the experiment. Data were expressed as the duration of tonic hind-limb extension (THE) after electroshock-induced seizure.

### 2.5 Treatments

Three experimental models of convulsion including intravenous and intraperitoneal administration of PTZ and electroshock were used to assess the seizure susceptibility of dairy products (Dough (0.5% fat), low-fat yogurt (1.3 % fat), high-fat yogurt (4% fat), low-fat milk (1.5% fat), high-fat milk (3% fat) and cheese (% 40 fat in dried material). For evaluation of acute experiment, dairy products and solvent (as a control) were administeredper orally through gavage in a volume of 0.1 ml/10g body weight of mice, 1 h before the administration of PTZ or electroshock and for evaluation of chronic effects, dairy products and solvent administered as a same dose for 7 days, and the last dose was administered 1 h before the administration of PTZ or electroshock on day 7.

### 2.6 Statistical analysis

Data are expressed as mean ± SEM. The one-way analysis of variance (ANOVA) followed by Dunnett test was employed to analyze the data. In order to determine the protective effects of dairy products against tonic seizures and death, Fisher's exact test was used. Statistical software was SPSS (Ver.18) and *P* < 0.05 was considered statistically significant.

## 3 Results

### 3.1 The effects of dairy products on intraperitoneal PTZ-induced seizure

**Fig. 1:**
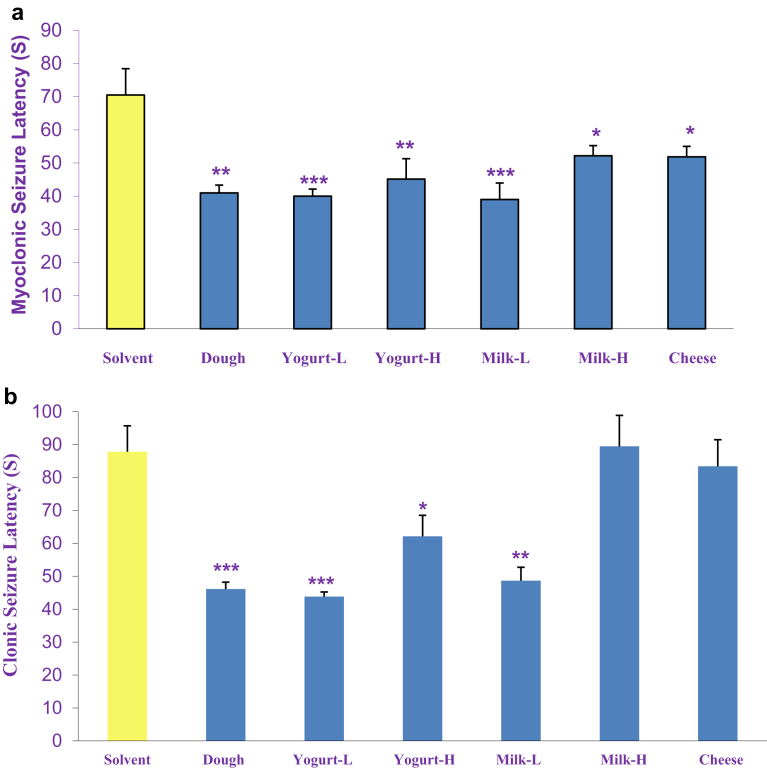
The acute effects of dairy products (Dough, low and high fat yogurt and milk and cheese, p.o.) on latency time to myoclonic seizure (a) and clonic seizure (b) induced by intraperitoneal PTZ infusion. Data are presented as mean ± S.E.M. *P < 0.05,**P < 0.01 and, ***P < 0.001 compared with the solvent group.

The results of acute effect of dairy products (Dough, low and high fat yogurt and milk and cheese, p.o.) on latency time to myoclonic seizure illustrated in Fig. 1A. One-way ANOVA revealed that all dairy products significantly decreased latency time to myoclonic seizure compared to the solvent group. Fig. 1B shows the effects of acuteadministration of dairy products (Dough, low and high fat yogurt and milk and cheese, p.o.) on latency time to clonic seizure. Statistical analysis revealed that all dairy products except high-fat milk and cheese significantly decreased latency time to clonic seizure compared to the solvent group.

In evaluation of the effect of acute administration of dairy products (Dough, low and high fat yogurt and milk and cheese, p.o.) on tonic seizure and mortality protection, Fisher's exact test demonstrated that Dough is the only dairy product which increased tonic seizure and mortality compared to the solvent group (Table 1).

**Table 1:** Effect of acute administration of dairy products (Dough, low and high fat yogurt and milk and cheese, p.o.) on tonic seizure and mortality protection in intraperitoneal PTZ-induced seizure model.

Groups	Tonic seizure protection (%)	Mortality protection (%)
Solvent	66.7	66.7
Dough	0 *	0 *
Yogurt-L	33.33	33.33
Yogurt-H	14.3	57.1
Milk-L	33.33	50
Milk-H	50	66.7
Cheese	42.9	57.1

Percentage of protection against incidence of tonic seizures and death subsequent intraperitoneal pentylenetetrazole was compared with solvent group using Fisher-exact test (*P < 0.05 compared to solvent group). L: Low fat and H: High fat.

**Fig. 2:**
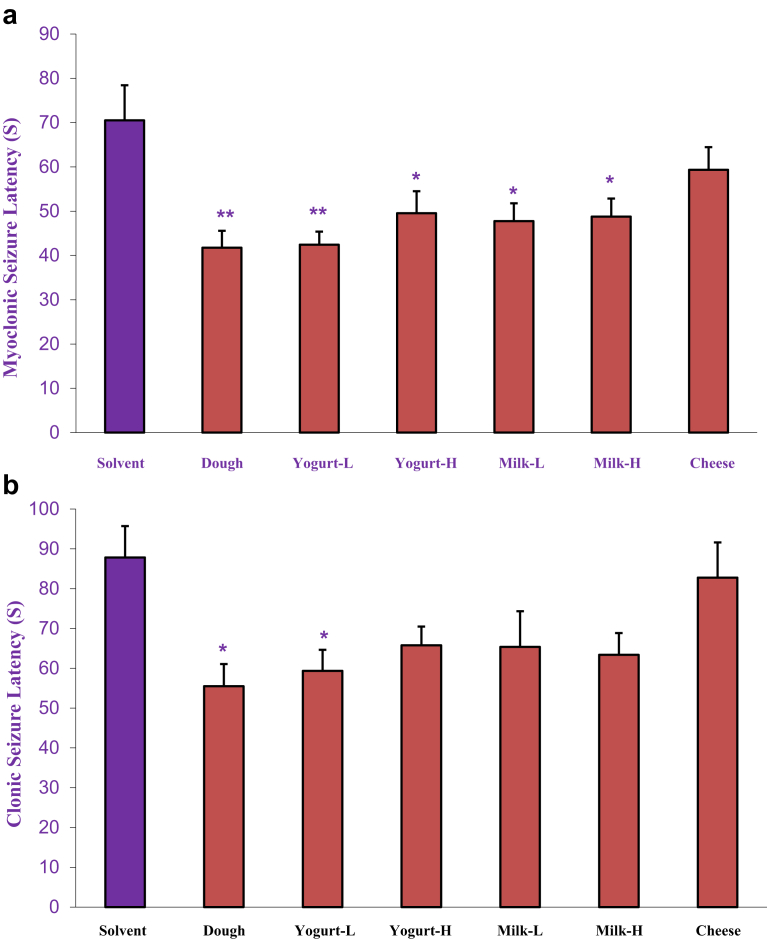
The chronic effects of dairy products (Dough, low and high fat yogurt and milk and cheese, p.o.) on latency time to myoclonic seizure (a) and clonic seizure (b)induced by intraperitoneal PTZ infusion. Data are presented as mean ± S.E.M. *P < 0.05 and **P < 0.01 compared with the solvent group.

The findings of the effects of chronic administration of dairy products (Dough, low and high fat yogurt and milk and cheese, p.o.) on latency time to myoclonic seizure are shown in Fig. 2A. One-way ANOVA revealed that all dairy products except cheese significantly decreased latency time to myoclonic seizure compared to the solvent group.

Fig. 2B shows the effects of chronic administration of dairy products (Dough, low and high fat yogurt and milk and cheese, p.o.) on latency time to clonic seizure compared to control group. Statistical analysis revealed that low-fat yogurt and Dough significantly decreased latency time to clonic seizure compared to the solvent group.

In order to find the result of chronic administration of dairy products (Dough, low and high fat yogurt and milk and cheese, p.o.) on tonic seizure and mortality protection, Fisher's exact test was done. This test revealed that all dairy products significantly increased tonic seizure compared to solvent group (Table 2).

**Table 2:** Effect of chronic administration of dairy products (Dough, low and high fat yogurt and milk and cheese, p.o.) on tonic seizure and mortality protection in intraperitoneal PTZ-induced seizure model.

Groups	Tonic seizure protection (%)	Mortality protection (%)
Solvent	66.7	66.7
Dough	0 *	25
Yogurt-L	11.1 *	66.7
Yogurt-H	11.1 *	77.8
Milk-L	0 *	62.5
Milk-H	0 *	50
Cheese	0 *	55.6

Percentage of protection against incidence of tonic seizures and death subsequent intraperitoneal pentylenetetrazole was compared with solvent group using Fisher-exact test (*P < 0.05 compared to solvent group). L: Low fat and H: High fat.

### 3.2 The effects of dairy products on intravenous PTZ-induced seizure

**Fig. 3:**
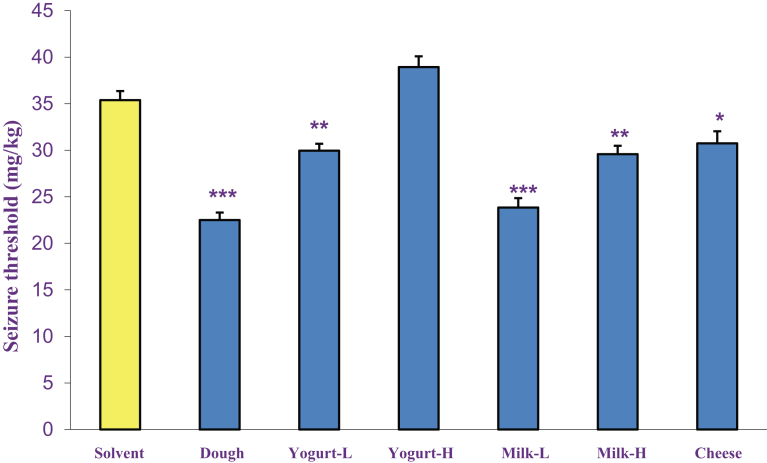
The acute effects of dairy products (Dough, low and high fat yogurt and milk and cheese, p.o.) on intravenous PTZ-induced clonic seizure threshold. Data are presented as mean ± S.E.M. *P < 0.05,**P < 0.01 and ***P < 0.001 compared with the solvent group.

**Fig. 4:**
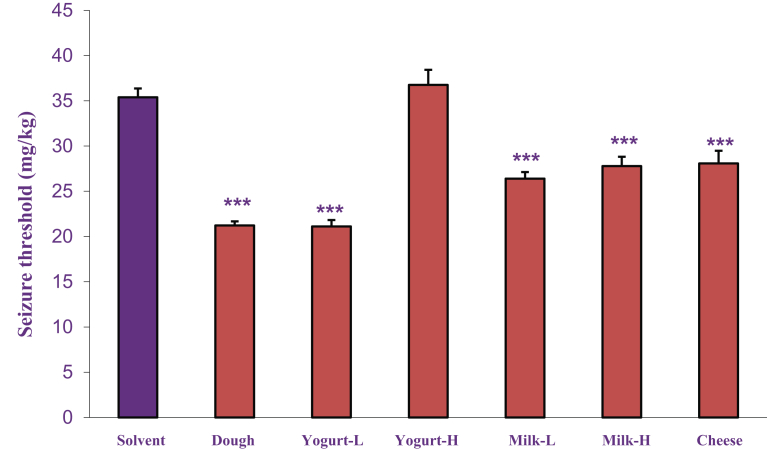
The chronic effects of dairy products (Dough, low and high fat yogurt and milk and cheese, p.o.) on intravenous PTZ-induced clonic seizure threshold. Data are presented as mean ± S.E.M.***P < 0.001 compared with solvent group.

The effects of acute and chronic administration of dairy products (Dough, low and high fat yogurt and milk and cheese, p.o.) on intravenous PTZ-induced clonic seizure threshold are shown in Figs. 3 and 4 respectively. Results revealed that all dairy products except high-fat yogurt, significantly decreased seizure threshold in comparison to the solvent group in both acute and chronic administration.

### 3.3 The effects of dairy products on electroshock-induced seizure

**Fig. 5:**
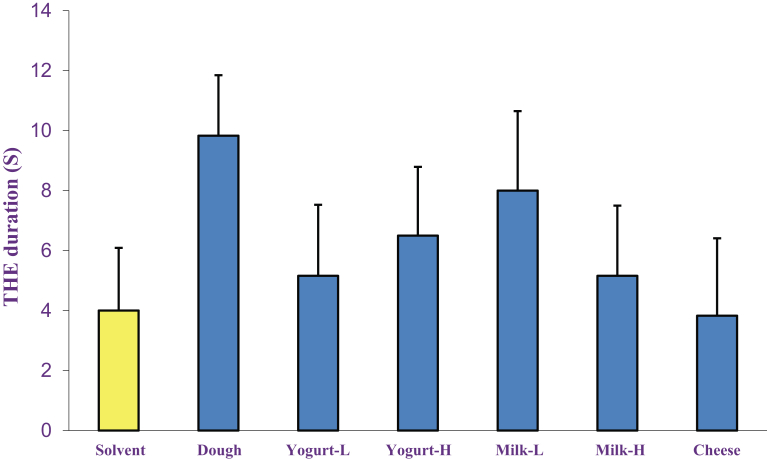
The acute effects of dairy products (Dough, low and high fat yogurt and milk and cheese, p.o.) on THE duration induced by electroshock. Data are presented as mean ± S.E.M.

**Fig. 6:**
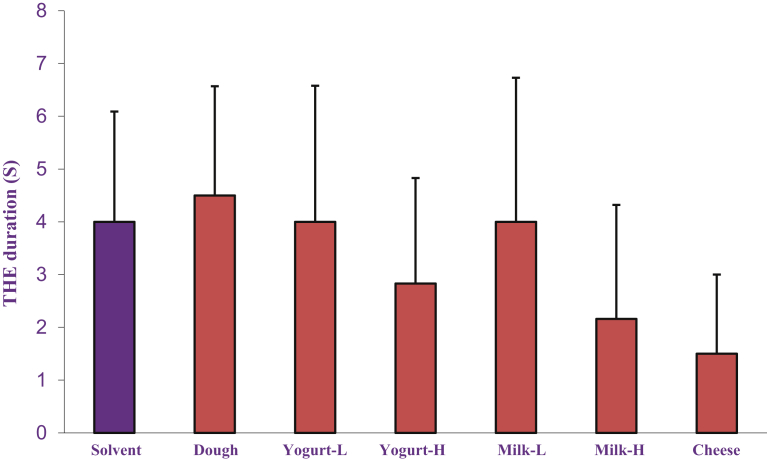
The chronic effects of dairy products (Dough, low and high fat yogurt and milk and cheese, p.o.) on THE duration induced by electroshock. Data are presented as mean ± S.E.M.

Figs. 5 and 6 show the effects of acute and chronic administrations of dairy products (Dough, low and high fat yogurt and milk and cheese, p.o.) on electroshock-induced seizure. One-way ANOVA illustrated that both acute and chronic administration of dairy products had no significant effects on THE duration induced by electroshock.

## 4 Discussion

Epilepsy is a common neurological disorder among children which has a strong effect on their development [13]. In recent decades, the hypothesis of certain foods or allergens involvement in convulsion has been proposed [3, 14, 15, 16]. Some foods may lower the seizure threshold or interact with prescribed medications [17]. The direct association between diet and epilepsy discovered accidentally. While scientists evaluated the role of special foods elimination on the treatment of hyperactivity disorder, they noticed that seizure frequency among children who had epileptic seizure previously remained low and interestingly patients became symptom-free after a while [18, 19]. Other studies report that food additives such as propionic acid induced convulsion in both human and rats or Monosodium glutamate (MSG) type additives can cause seizure in rats [4, 5, 6]but to our knowledge there are limited experimental studies for evaluation of dairy products effects on convulsion threshold so the aim of present study was to evaluate the acute and chronic effects of different dairy products administration such as Dough, high and low-fat yogurt, high and low-fat milk and cheese on seizure activity. According to our results, acute and chronic administration of all dairy products except high-fat yogurt decreased seizure threshold in intravenous PTZ-induced seizure. Furthermore, in intraperitoneal PTZ model, acute and chronic administration of dairy products (except cheese in chronic administration) reduced time latencies to the onset of myoclonic jerk and generalized seizure. Data analysis of acute administration of dairy products illustrated that all of them except cheese and high-fat milk decreased time latencies to the onset of clonic jerk and in chronic administration, Dough and low-fat yogurt are the only products which lessened clonic jerk time latencies in intraperitoneal PTZ model. In summation, the results of present study indicated that dairy products with lower fat significantly reduced seizure threshold and myoclonic or clonic jerk latencies in intravenous and intraperitoneal PTZ-induced seizure respectively but high-fat products or cheese had lower impact on seizure activity. Indeed cow's milk protein allergy was documented in several studies [7]. Although the exact mechanism of provoking seizure attack by certain food is unknown but some studies emphasizes on allergic hypothesis. Casein is an important protein of milk which may be very difficult to digest and often cause hypersensitivity [20]. It seems the release of inflammatory cytokinesduring the process of immune system response which alter the blood-brain-barrier (BBB) integrityand activation of brain mast cellslead to neuronal inflammation which could constitute on epileptogenic focus [8]. It suggests that it might be an association between epilepsy and food hypersensitivity. In a new research, Albenzio and colleagues have focused on the main role of milk protein fractions in inducing of cytokines and oxidative stress responses. Indeed the current investigation was an ex-vivo study which pro and anti-inflammatory cytokines and oxidative status in cultured peripheral blood mononuclear cells (PBMC) isolated from epileptic children in the response of diverse protein fractions (from different milking species) were evaluated. The results of study have shown that milk protein components may lead to immune response in children with generalized epilepsy and difference in cytokine responses may be associated with genetic polymorphism of milk proteins [21].

On the other hand, the level of aminoacid-derived neurotransmitters in the brain may be affected by dietary precursors [9]. For example, the patterns of plasma aminoacids such as tryptophan and tyrosine may be altered by special diet and fluctuation of them in the brain affects synthesis of some neurotransmitters such as serotonin and catecholamine by neurons. Furthermore,opiate-like peptides have been found in different foods such as milk. Actually, peptidergic substances which have strong effects on brain metabolism play a crucial role in pathophysiology of epilepsy [9]. Moreover, Ismail et al. have shown that brain-derived neurotrophic factor (BDNF) content in serum of epileptic infants and their mother's milk are higher than control group [22]. BDNF is a small protein belongs to neurotrophin family which is expressed in mammalian brain especially in hippocampus and cerebral cortex. It plays an important role in neuronal proliferation [23, 24]. Several invivo and invitro studies have demonstrated that acute administration of BDNF increases neuronal excitability [25, 26]. It seems that BDNF may facilitate epileptogenesis and higher level of BDNF in epileptic infants' mother milk may be one of possible mechanism of seizure induction [27, 28]. Unfortunately, we could not find any information about BDNF content of cow's milk.

Previous experiments have shown that high-fat (ketogenic) diet elevate seizure threshold and lead to the reduction in the seizure frequencyso the clinical administration of them are used as a treatment of epilepsy for over 75 years [29, 30]. Neuroprotective and disease-modifying effects of them were explained in the other neurological disorders like Parkinson's disease and Alzheimer's disease [31]. For explanation of beneficial effect of high fat diet on seizure, different mechanisms have been proposed. For example, neurons become more resilient in face of metabolic demands as a result of changes in ATP production or the change in brain pH alters the neuronal excitability. The fatty acids and ketone bodies may have inhibitory effect on ion channels and aminoacid metabolism will shift to the synthesis of the inhibitory neurotransmitter GABA [32].

These findings besides other studies which represent that full-fat milk or higher fat dairy products as an effective anti-inflammatory factor through modulation of cytokine gene expression and reduction of oxidative stress [33, 34]may explain our findings which showthat high fat dairy products decrease seizure activity. On the other hand, Alzoubi and colleagues claim that prolonged consumption of high fat dietmay increase susceptibility to PTZ-induced seizures through induction of oxidative stress in the brain which their findings are in contrast with our results [35].

Moreover, the results of present study showed that both acute and chronic administration of dairy products had no effect on seizure induced by electroshock. Different experimental models are employed to evaluate the effect of diverse products on convulsion and those induced by pentylenetetrazole (PTZ) or electroshock stimuli are commonly used [36]. PTZ bind to benzodiazepine and picrotoxin sites of GABA_A_ receptor complex and inhibit receptor activity which is essential in seizure inhibition [12]. This model is used for induction of generalized absent seizure while electroshock-induced seizure considered as an animal model of human clonic and/or tonic generalized convulsion. These two models examine different aspects of seizure. The PTZ-induced seizure tests the threshold to seizure onset and the other evaluates the propagation of an induced seizure. Respect to seizure severity and seizure threshold, these two different models may reveal different results [37]. According to the results of Bough and his colleagues, a ketogenic diet had various impacts on seizure induced by electroshock and PTZ. They found that ketogenic diet developed seizures in response to electroshock but elevated seizure thresholds conversely when tested by PTZ infusion [38]or pharmacological study using PTZ and electroshock revealed that administration of nimodipine (a calcium channel blocker) conferred protection from PTZ-induced seizure but had no effect on electroshock-induced seizure [39]. Hence, diet-related seizures may depend upon the method which seizures are provoked.

## 5 Conclusion

We believe that our study is novel because it is the first time that the effects of dairy products on diverse experimental models of seizures were evaluated. The results of study demonstrated that low-fat dairy products had strong impact on increasing seizure activity on PTZ-induced seizure compared to higher fat dairy products which may be as a result of inflammatory response induced by lower fat dairy foods. It seems that inconformity between observed results of PTZ and electroshock-induced seizures might be as a consequence of different types of seizure induced by these two experimental models.

## Declarations

### Author contribution statement

Soroor Inaloo: Conceived and designed the experiments, Wrote the paper.

Fatema Pirsalami: Performed the experiments.

Mona Dastgheib: Contributed reagents, materials, analysis tools or data; Wrote the paper.

Leila Moezi: Conceived and designed the experiments; Analyzed and interpreted the data; Contributed reagents, materials, analysis tools or data; Wrote the paper.

### Funding statement

This work was supported by Shiraz University of Medical Sciences grants No: 95-01-36-13167.

### Competing interest statement

The authors declare no conflict of interest.

### Additional information

No additional information is available for this paper.
